# *Slc6a3*-dependent expression of a CAPS-associated *Nlrp3* allele results in progressive behavioral abnormalities and neuroinflammation in aging mice

**DOI:** 10.1186/s12974-020-01866-6

**Published:** 2020-07-17

**Authors:** Katharine M. von Herrmann, Faith L. Anderson, Eileen M. Martinez, Alison L. Young, Matthew C. Havrda

**Affiliations:** Department of Molecular and Systems Biology, Geisel School of Medicine at Dartmouth College, 1 Rope Ferry Road, Hanover, NH 03755 USA

**Keywords:** Neuroinflammation, NLRP3 inflammasome, Astrogliosis, Cryopyrin-associated periodic syndromes

## Abstract

**Background:**

An association between neuroinflammation and age-related neurologic disorders has been established but the molecular mechanisms and cell types involved have not been thoroughly characterized. Activity of the proinflammatory NLRP3 inflammasome is implicated in Alzheimer’s and Parkinson’s disease and our recent studies in patients suggest that dopaminergic neurons within the degenerating mesencephalon express NLRP3 throughout the progression of PD. Here, we directly test the impact of enhanced inflammasome activity in mesencephalic neurons by characterizing motor function, tissue integrity, and neuroinflammation in aging mice harboring hyperactivating mutations within the endogenous murine *Nlrp3* locus, enabled only in cells expressing the dopaminergic neuron-specific *Slc6a3* promoter.

**Methods:**

We compared mice harboring inducible alleles encoding the cryopyrin-associated periodic syndrome activating mutations *Nlrp3*^*A350V*^ and *Nlrp3*^*L351P*^ inserted into the endogenous mouse *Nlrp3* locus. Tissue specific expression was driven by breeding these animals with mice expressing Cre recombinase under the control of the dopaminergic neuron-specific *Slc6a3* promoter. The experimental mice, designed to express hyperactive NLRP3 only when the endogenous mouse *Nlrp3* promotor is active in dopaminergic neurons, were analyzed throughout 18 months of aging using longitudinal motor function assessments. Biochemical and histologic analyses of mesencephalic tissues were conducted in 1- and 18-month-old animals.

**Results:**

We observed progressive and significant deficits in motor function in animals expressing *Nlrp3*^*L351P*^, compared with animals expressing *Nlrp3*^*WT*^ and *Nlrp3*^*A350V*^. Age-dependent neuroinflammatory changes in the mesencephalon were noted in all animals. Analysis of GFAP-immunoreactive astrocytes in the substantia nigra revealed a significant increase in astrocyte number in animals expressing *Nlrp3*^*L351P*^ compared with *Nlrp3*^*WT*^ and *Nlrp3*^*A350V*^. Further analysis of *Nlrp3*^*L351P*^ striatal tissues indicated genotype specific gliosis, elevated *Il1b* expression, and both morphologic and gene expression indicators of proinflammatory A1 astrocytes.

**Conclusions:**

Dopaminergic neurons have the potential to accumulate NLRP3 inflammasome activators with age, including reactive oxygen species, dopamine metabolites, and misfolded proteins. Results indicate the *Nlrp3* locus is active in dopaminergic neurons in aging mice, and that the hyperactive *Nlrp3*^*L351P*^ allele can drive neuroinflammatory changes in association with progressive behavioral deficits. Findings suggest neuronal NLRP3 inflammasome activity may contribute to neuroinflammation observed during normal aging and the progression of neurologic disorders.

## Background

Inflammasomes are a class of cytosolic multi-protein complexes whose activation results in intracellular cascades that initiate and propagate proinflammatory signaling [1]. Sustained inflammasome activation can lead to pyroptosis, a subcategory of programmed cell death [2, 3]. Inflammasome complexes are categorized based on the presence of pattern recognition receptors belonging to either the nucleotide-binding domain-like receptors (NLRs), absent in melanoma 2-like receptors (ALRs), or pyrin families [4, 5]. Upon activation, NLRP3 inflammasome proteins form a complex with ASC and pro-caspase-1, inducing maturation of caspase-1, followed by cleavage of proinflammatory cytokines including pro-interleukin-1 beta (pro-IL-1β) and pro-IL-18, and the pore-forming protein gasdermin D [6–9]. Proteolytically processed cytokines are released from the cell and can propagate inflammation in the surrounding tissue. The understanding of inflammasome biology was aided by the identification of rare familial syndromes including familial cold autoinflammatory syndrome (FCAS), Muckle-Wells syndrome (MWS), and neonatal onset multisystem inflammatory disease (NOMID) [10, 11]. These hereditary cryopyrin-associated periodic syndromes (CAPS) are typified by chronic inflammatory symptomology caused by point mutations in the NLR family gene *NLRP3*, resulting in the production of a hyperactive NLRP3 protein [12, 13]*.*

Inflammasomes are widely accepted to be key mediators of the innate immune response with many studies characterizing their activities in hematopoietic cells of the myeloid lineage, and more recent studies by our laboratory [14] and others [15–19] characterizing NLRP3 inflammasome activity in functionally related microglia of the central nervous system (CNS). Increasing evidence indicates that the inflammasome stress-sensing pathway may be utilized by a host of other cell types. NLRP3 inflammasome activity in hepatocytes is a key feature of liver disease [20], observable in animals exposed to high-fat diets [21] as well as in tissues obtained from patients with non-alcoholic fatty liver disease [22]. *NLRP3* polymorphisms are associated with an elevated risk of developing Crohn’s disease [23, 24]. Subsequent studies indicate significant *NLRP3* expression and inflammasome activity in intestinal epithelial cells that influence inflammatory bowel disease progression [25, 26]. Sustained *NLRP3* expression was observed in distressed neurons surrounding ischemic stroke [27] as well as in primary cortical neurons in an experimental traumatic brain injury (TBI) model [28]. Expression of the inflammasome family pattern recognition receptors *NLRP1* and *AIM2* has also been reported in neurons in mouse and rat models respectively [29, 30]. In humans, *NLRP1* expression was observed in neurons of patients with spinal cord injury [31], and in primary human neuron cultures [32]. Collectively, these data, among others, indicate that a variety of cell types utilize the inflammasomes to participate in the innate immune response.

A growing body of research has characterized the activity of intracellular inflammasomes in age-related neurodegenerative disorders based on the widely held belief that these disorders are exacerbated by chronic neuroinflammation [19, 33, 34]. The NLRP3 inflammasome is of particular interest in neurologic disorders because it can be activated by sterile cellular stressors common to many neurodegenerative diseases including proteinaceous insult [35], oxidative stress [36, 37], environmental toxicants [38], and cell death [39]. Characterizing NLRP3 in Parkinson’s disease (PD), we observed NLRP3 inflammasome activity in PD patients [40] and demonstrated that loss of *Nlrp3* is neuroprotective in a toxicant-based mouse model of PD [14]. We also identified a single-nucleotide polymorphism in the *NLRP3* exome sequence associated with a reduced risk of PD that we observed to negatively impact NLRP3 protein homeostasis in vitro [40]. These studies, and others [17, 41, 42], of NLRP3 activity in PD are consistent with similar studies of NLRP3 in Alzheimer’s disease (AD) [43–46], suggesting that the NLRP3 inflammasome is a core proinflammatory mediator in neurodegenerative disorders. NLRP3 activity in neurologic disorders is readily observed in microglia [47] but our studies also revealed NLRP3 expression in surviving dopaminergic (DA) neurons of the mesencephalon in post-mortem tissues obtained from PD patients [40]. Further analysis corroborated our patient data, indicating inducible expression of *NLRP3* in tyrosine hydroxylase-positive differentiated Lund human mesencephalic (LUHMES) neurons in culture and evidence of NLRP3 inflammasome activity in differentiated, human SH-SY5Y cells. Our data indicating NLRP3 expression and activity in DA neurons are consistent with reports of other inflammasome-related pattern recognition receptor expression in neurons described above, yet, to our knowledge, no studies have directly tested the consequence of neuronal inflammasome activity throughout the process of aging.

To characterize the effect of enhanced NLRP3 inflammasome activity in mesencephalic neurons in the context of aging, we bred mice harboring well-characterized, inducible *Nlrp3* alleles engineered to contain hyperactivating polymorphisms associated with CAPS [48] with mice expressing Cre recombinase under the control of the *Slc6a3* promoter, encoding the DA neuron-specific dopamine transporter. These “knock-in” mice are designed to express hyperactive NLRP3 in a Cre-dependent manner only when the endogenous *Nlrp3* promoter is active, allowing us to determine the result of enhanced NLRP3 inflammasome activity occurring exclusively when the *Nlrp3* gene is active in *Slc6a3-*expressing neurons, throughout aging. Longitudinal behavioral analyses indicate that tissue-specific expression of the *Nlrp3*^*L351P*^ polymorphism resulted in significant age-dependent motor deficits as compared with the *Nlrp3*^*A350V*^ polymorphism and mice expressing the WT allele. Post-mortem studies indicate enhanced neuroinflammation specifically in tissues obtained from animals expressing *Nlrp3*^*L351P*^. Our collective data indicate that the *Nlrp3* locus is active in DA neurons and that enhanced neuronal inflammasome activity is sufficient for the development of more widespread neuroinflammatory changes associated with motor deficits and advanced age.

## Methods

### Animals

**Fig. 1:**
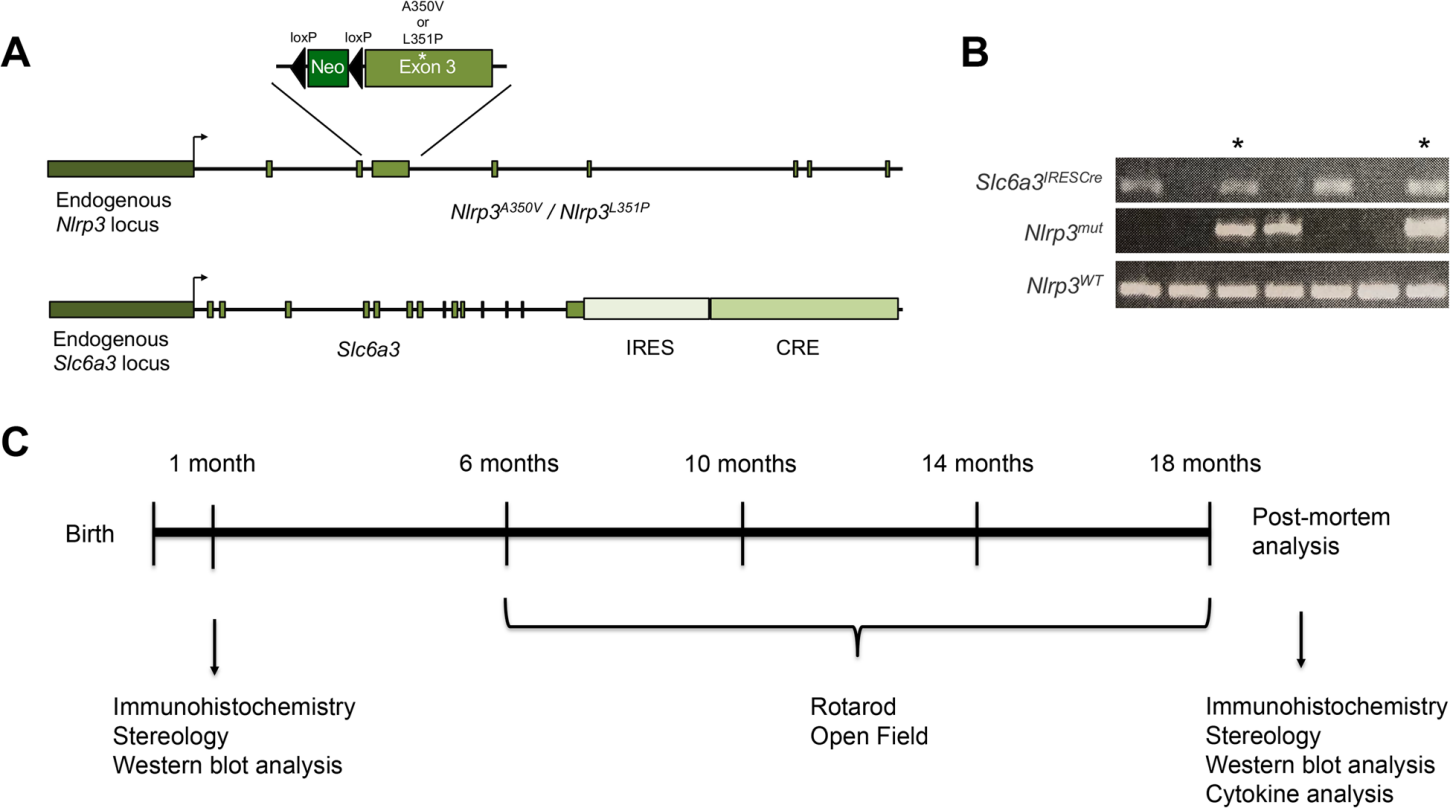
Longitudinal model of mice expressing hyperactive *Nlrp3* mutations under the endogenous promotor in DA neurons. **a** Transgenic mice harboring Cre-driven genetic mutations in *Nlrp3*, either *Nlrp3*^*A350V*^, or *Nlrp3*^*L351P*^, previously associated with the cryopyrin-associated periodic syndromes, were bred with mice harboring IRES-Cre under the control of the *Slc6a3* promotor. **b** Representative genotyping with PCR-based detection of *Nlrp3*^*WT*^ and *Nlrp3*^*mut*^ alleles. Mice double positive for *Slc6a3*^*IRESCre*^ and *Nlrp3*^*mut*^ (*Nlrp3*^*A350V*^ or *Nlrp3*^*L351P*^) were included in the study as indicated by *, as well as *Nlrp3*^*WT/WT*^ mice as controls. All mice contain one background copy of the *Nlrp3*^*WT*^ gene. **c** Experimental design of longitudinal study. Histologic sections were analyzed at both 1 month and 18 months of age, and behavior analysis was conducted at the 6, 10, 14, and 18-month time points

Animal experiments were approved by the Dartmouth Institutional Animal Care and Use Committee and conducted in accordance with federal guidelines pertaining to animal research. All mouse experiments were approved by the Institutional Animal Care and Use Committee (IACUC) at Dartmouth protocol entitled “Animal Models of Neurodegenerative Diseases,” protocol no. 00002117. Mice harboring *Nlrp3* mutant alleles were purchased from Jackson Laboratories (Bar Harbor, ME), developed and characterized as previously described [48]. MWS-associated *Nlrp3*^*A350VneoR*^ (stock no. 017969) and FCAS-associated *Nlrp3*^*L351PneoR*^ (stock no. 017970) alleles were generated in the endogenous mouse *Nlrp3* gene preceded by a reverse oriented neomycin cassette flanked by loxP sites. The mouse *Nlrp3* allele is functionally null in both animals prior to Cre recombinase-mediated deletion of the neomycin cassette. We bred both strains to commercially available mice expressing Cre recombinase in cells expressing the dopamine transporter gene, *Slc6a3*^*IRESCre*^ (stock no. 006660). The *Slc6a3*^*IRESCre*^ strain is now convincingly validated and in wide use [49–51], originally characterized using the ROSA reporter line to express CRE specifically within neuronal populations of the mesencephalon [52]. This process generated mice that selectively express the hyperactive *Nlrp3* alleles only when the endogenous murine *Nlrp3* gene is expressed in dopaminergic neurons. Mice were heterozygous for the hyperactive *Nlrp3* mutant alleles, and were engineered to retain one copy of *Nlrp3*^*WT*^ in all cells of the animal. The breeding resulted in three distinct cohorts of animals: *Nlrp3*^*WT*^*/Slc6a3*^*WT*^, *Nlrp3*^*A350V*^*/Slc6a3*^*IRESCre*^, and *Nlrp3*^*L351P*^*/Slc6a3*^*IRESCre*^. Genotyping was conducted to validate all mice included in the study (Fig. 1b). Primers were obtained from Integrated DNA Technologies (Coralville, IA) (Table 1).

**Table 1:** Primers used for animal genotyping and gene expression analysis

Gene	Forward primer	Reverse primer
*Nlrp3*^*WT*^	CACCCTGCATTTTGTTGTTG	CGTGTAGCGACTGTTGAGGT
*Nlrp3*^*mutant*^	GCTACTTCCATTTGTCACGTCC	CGTGTAGCGACTGTTGAGGT
*Slc6a3*^*WT*^	TGGCTGTTGGTGTAAAGTGG	GGACAGGGACATGGTTGACT
*Slc6a3*^*IRESCre*^	TGGCTGTTGGTGTAAAGTGG	GGCCCTCACATTGCCAAAAG
*C3*	CCAGCTCCCCATTAGCTCTG	GCACTTGCCTCTTTAGGAAGTC
*Ggta1*	GTGAACAGCATGAGGGGTTT	GTTTTGTTGCCTCTGGGTGT
*H2-D1*	TCCGAGATTGTAAAGCGTGAAGA	ACAGGGCAGTGCAGGGATAG
*Il1b*	CGCAGCAGCACATCAACAAGAGC	TGTCCTCATCCTGGAAGGTCCACG
*Actb*	GCACCACACCTTCTACAATGAGC	ATGTCACGCACGATTTCCC

### Gene expression analysis

To validate expression of engineered *Nlrp3* alleles, DA neuron terminal-rich striatal tissues were harvested from 18-month-old animals and total mRNA was isolated using RNeasy Mini Kit according to the manufacturer’s instructions (Qiagen, Hilden, Germany). Total mRNA was reversed transcribed with iScript cDNA Synthesis Kit (Bio-Rad Laboratories, Hercules, CA). *Nlrp3* transcripts were amplified and sequenced with Sanger sequencing techniques conducted by Dartmouth’s Molecular Biology Shared Resource. Similar methods were utilized for real-time PCR analysis of *C3*, *Ggta1*, *H2-D1*, and *Il1b* transcripts. Analysis was completed using the CFX96 Touch Real-Time PCR Detection System (Bio-Rad Laboratories, Hercules, CA) with SYBR Green reagent (Bio-Rad Laboratories, Hercules, CA). All primers were obtained from Integrated DNA Technologies (Coralville, IA) (Table 1).

### Behavioral analysis

Behavior was analyzed longitudinally using the rotarod and open field paradigms at 6, 10, 14, and 18 months of age as previously reported [14]. Rotarod training and testing sessions consisted of two 300-s trials separated by 15-min intervals. Recorded data for each animal captured the time each mouse spent on the rotarod, with a maximum of 300 s. Rotarod testing was performed on the fourth day following three consecutive training days. Open field testing was conducted to assess behavior and general motor activities using a computer-assisted photobeam detection system (TruScan, Thermo Fisher Scientific, Waltham, MA). Data were collected throughout the 30-min session. Measurements analyzed in this study include move time (s), rest time (s), distance moved (cm), total velocity (cm/30 min), average velocity (cm/min), and center time (s). All behavior data were analyzed with the GraphPad Prism software (San Diego, CA).

### Immunohistochemistry and stereology

Immunohistochemistry and stereology experiments were performed as previously described [14]. Briefly, brain tissues were isolated and dissected. One hemisphere was submitted for sectioning and staining (Neuroscience Associates, Knoxville, TN). Sections were cut at 60 μM, stained with anti-tyrosine hydroxylase antibodies**,** and then counterstained with Nissl. Unbiased stereology was conducted using the optical fractionator method (Stereo Investigator, Microbrightfield, Williston, VT), counting every fourth section of the SNpC (5 sections/animal). Remaining tissues were embedded for either paraffin or cryosectioning and sliced at 5 μM. Immunohistochemical studies were performed at the Dartmouth Research Pathology Shared Resource using an automated staining system (Leica Bond Max, Buffalo Grove, IL). Immunofluorescence studies were conducted manually on paraffin sections using a sodium citrate epitope retrieval protocol. Primary antibodies used in this study include the following: ionized calcium**-**binding adaptor molecule 1 (IBA1) (NB100-1028, Novus Biologicals, Centennial, CO), glial fibrillary acidic protein (GFAP) (2021-05, Agilent Technologies, Santa Clara, CA), apoptosis-associated speck-like protein containing a CARD (ASC) (AL177, AdipoGen Life Science, San Diego, CA), interleukin-1 beta (IL-1β) (AF-401-NA, R&D Systems, Minneapolis, MN), and tyrosine hydroxylase (TH) (P40101, Pel-Freez Biologicals, Rogers, AR). IHC studies were conducted with either BOND polymer refined detection system (Leica Biosystems, Wetzlar, Germany) or HRP Polymer (Biocare Medical, Pacheco, CA). Images were obtained using an Olympus BX51 microscope. Confocal microscopy studies were conducted with fluorescently labeled secondary antibodies raised in donkey (Alexa Fluor 488 ab150061, Alexa Fluor 555 ab150130, Abcam, Cambridge, UK). Imaging was conducted using a Zeiss LSM 800 laser point scanning microscope under the guidance of Dartmouth’s Microscopy**—**Irradiation, Pre-clinical Imaging**,** and Microscopy Shared Resource.

### SDS-PAGE and western blotting

SDS-PAGE and western blotting experiments were performed as previously described [40]. Striatal tissues were isolated and homogenized in lysis buffer (150 mM NaCl, 50 mM Tris, 1% Triton) supplemented with protease and phosphatase inhibitors. Proteins were separated using the NuPAGE system on 4–12% pre-cast gradient gels and MES/SDS running buffer (Thermo Fisher Scientific, Waltham, MA) and then were transferred to Immobilon P transfer membrane (Millipore, Burlington, MA) using a Mini Blot Module (Thermo Fisher Scientific, Waltham, MA). Blots were probed with anti-tyrosine hydroxylase (TH) antibodies (MAB318, Millipore, Burlington, MA) and anti-actin HRP-coupled antibodies (A3854, Sigma-Aldrich, St. Louis, MO). HRP-conjugated anti-mouse secondary antibodies (Millipore, Burlington, MA) were used to detect TH immunoreactivity in association with chemiluminescence-based detection, ECL-Plus (Thermo Fisher Scientific, Waltham, MA).

### Statistical analysis

At least 3 biologic replicates were conducted for gene expression experiments, and data were analyzed with one-way ANOVA. All immunohistochemistry experiments were conducted by a blinded investigator, and quantified data were analyzed with one-way ANOVA or two-tailed Student’s *t* test as appropriate. Behavioral tests were run by an investigator blinded to genotype. Longitudinal rotarod and open field data were analyzed by two-way repeated measures ANOVA. ANOVA analyses were followed by Tukey’s post hoc multiple comparisons test when appropriate. Endpoint 18-month analysis was analyzed with one-tailed Student’s *t* test. Unbiased stereology was conducted by a single, blinded investigator. All statistical tests were conducted with the GraphPad Prism software (San Diego, CA).

## Results

Evidence of NLRP3 inflammasome protein expression in neurons has been reported by our laboratory and others [27, 40, 53–55]**;** however, the impact of neuronal inflammasome activity in aging animals has not been analyzed directly. Having identified NLRP3 expression in remaining DA neurons from PD patients and in human neuronal cell lines [40], we established a genetically engineered mouse model to characterize longitudinally the effect of enhanced DA neuron-specific NLRP3 inflammasome activity throughout aging. We took advantage of DA neuron-specific *Slc6a3*^*IRESCre*^ mice and animals originally reported by Brydges et al. [48] in which the endogenous *Nlrp3* locus was modified to encode CAPS-associated activating mutations A350V or L351P under the control of Cre recombinase (Fig. 1a) (see “Methods” section). The study animals were confirmed by genotyping (Fig. 1b), and fell into three cohorts: *Nlrp3*^*WT/WT*^*/Slc6a3*^*IRESCre*(*−*)^, *Nlrp3*^*WT/A350V*^*/Slc6a3*^*IRESCre*(*+*)^, *Nlrp3*^*WT/L351P*^*/Slc6a3*^*IRESCre*(*+*)^ referred to herein as *Nlrp3*^*WT*^, *Nlrp3*^*A350V*^, and *Nlrp3*^*L351P*^ respectively. Critically, this cross generates modifications in the endogenous murine *Nlrp3* locus [48], resulting in animals that will only express the hyperactive CAPS alleles when the endogenous *Nlrp3* promoter is active in DA neurons.

**Fig. 2:**
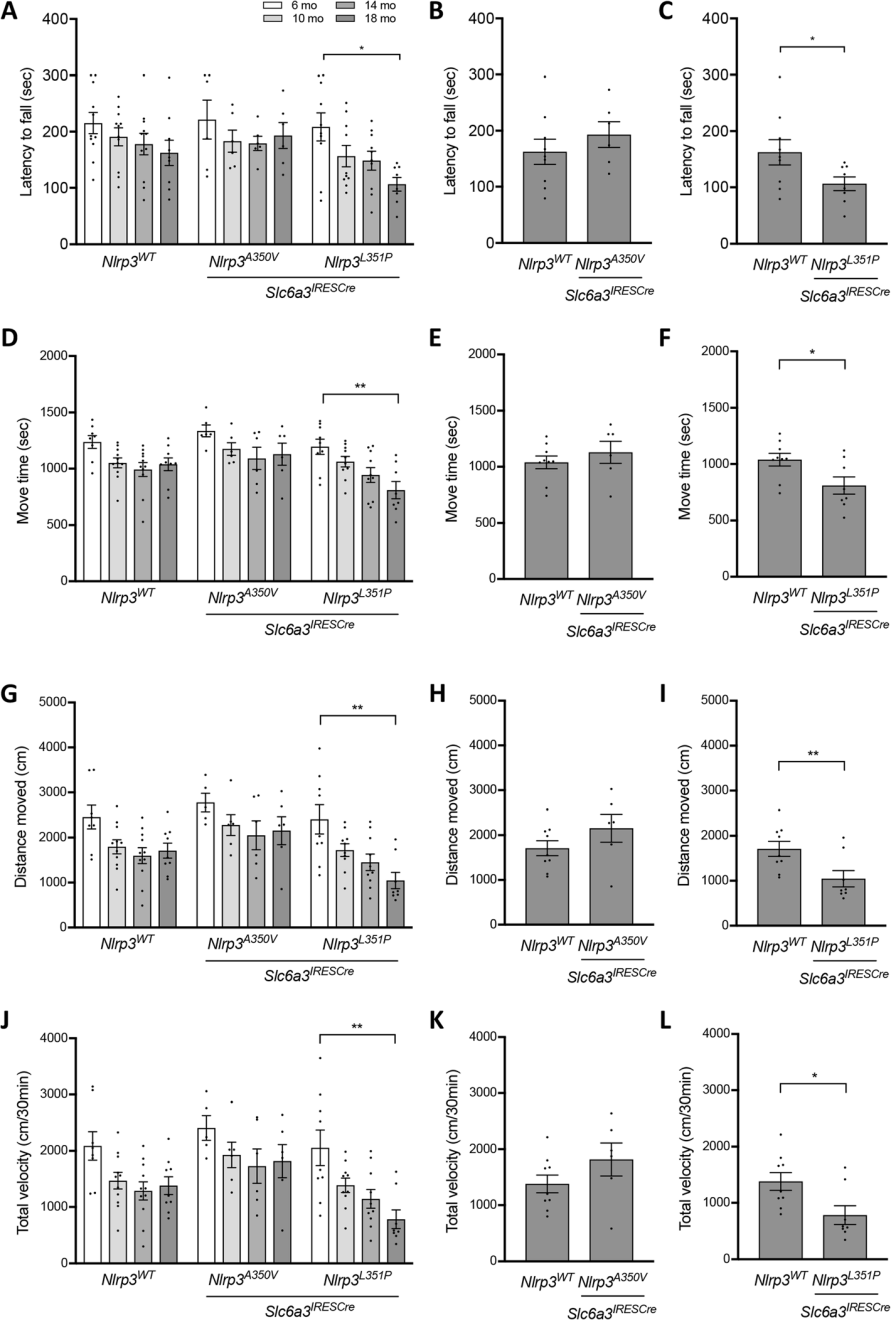
Behavioral analysis revealed significant reduction in motor function over time in *Nlrp3*^*L351P*^ cohort. Behavioral analyses were conducted at 6, 10, 14, and 18-month time points in cohorts (*Nlrp3*^*WT*^*n* = 11, *Nlrp3*^*A350V*^*n* = 6, *Nlrp3*^*L351P*^*n* = 9). **a***Nlrp3*^*L351P*^ mice had significant rotarod behavior deficits with age (two-way ANOVA, *p* = 0.0211 (genotype), *p* = 0.0052 (time); Tukey’s post hoc multiple comparisons test, * *p* = 0.02). **b**, **c** Endpoint analysis at 18 months shows *Nlrp3*^*L351P*^ expressing mice, not *Nlrp3*^*A350V*^, were significantly impaired compared to *Nlrp3*^*WT*^ (* *p* = 0.026, *t* test). In open field analysis, animals expressing *Nlrp3*^*L351P*^**d** moved for significantly less time (two-way ANOVA, *p* = 0.0018 (genotype), *p* < 0.0001 (time); Tukey’s post hoc multiple comparisons test, ** *p* = 0.0027), **g** moved a significantly shorter distance (two-way ANOVA, *p* = 0.0008 (genotype), *p* < 0.0001 (time); Tukey’s post hoc multiple comparisons test, ** *p* = 0.0015), and **j** traveled at a significantly lower velocity (two-way ANOVA, *p* = 0.0008 (genotype), *p* < 0.0001 (time); Tukey’s post hoc multiple comparisons test, ** *p* = 0.0020) at 18 months compared to 6 months of age. No significant changes were observed in the *Nlrp3*^*WT*^ and *Nlrp3*^*A350V*^ over time. Endpoint comparison at 18 months between cohorts revealed a significant decline in **f** move time (* *p* = 0.0135, *t* test), **i** distance moved (** *p* = 0.0085, *t* test), and **l** total velocity (* *p* = 0.01, *t* test) in animals expressing *Nlrp3*^*L351P*^ compared to *Nlrp3*^*WT*^ animals. **e**, **h**, **k** This difference was not observed in *Nlrp3*^*A350V*^ expressing animals when compared to controls. Error bars represent s.e.m.

Endogenous *Nlrp3* expression increases with aging in the CNS [56–59] prompting us to characterize the influence of the *Nlrp3* locus in DA neurons at advanced ages (Fig. 1c). We conducted longitudinal rotarod and open field analyses comparing behavior in *Nlrp3*^*WT*^, *Nlrp3*^*A350V*^, and *Nlrp3*^*L351P*^ expressing animals at 6, 10, 14, and 18 months of age. As expected, we observed a reduction in activity of older mice across all three genotypes (Fig. 2). *Nlrp3*^*L351P*^ animals had a progressive decline in rotarod performance, while there was no significant change observed over the same time period in the *Nlrp3*^*WT*^ and *Nlrp3*^*A350V*^ expressing cohorts (Fig. 2a). Endpoint analysis further confirmed the repeated measure findings indicating that the decreased latency to fall from the rotarod was specific to the *Nlrp3*^*L351P*^ animals (Fig. 2b, c). Open field analysis indicated that over the same 12-month time course, *Nlrp3*^*L351P*^ animals also had significantly reduced locomotor activity, spending shorter periods of time in motion (Fig. 2d), and moving significantly shorter distances (Fig. 2g) and at reduced velocities overall (Fig. 2j). Endpoint analyses for each of these measures again confirmed the longitudinal data indicating specific deficits in *Nlrp3*^*L351P*^ mice at 18 months of age (Fig. 2e, f, h, i, k, l). Analysis of rest time, average velocity, and time spent in the center of the open field chamber revealed a significant reduction in all genotypes throughout aging (Supplementary Figure 1). Comparing individual time points, *Nlrp3*^*L351P*^ expressing animals spent significantly less time moving at 18 months compared to 6 months and did so with a smaller velocity (Supplementary Figures 1A, B). These behavior assays reveal that animals expressing *Nlrp3*^*L351P*^ exhibit a significant reduction in motor function over time in the 18-month longitudinal study as compared with identically aged animals harboring *Nlrp3*^*WT*^ and *Nlrp3*^*A350V*^ alleles.

**Fig. 3:**
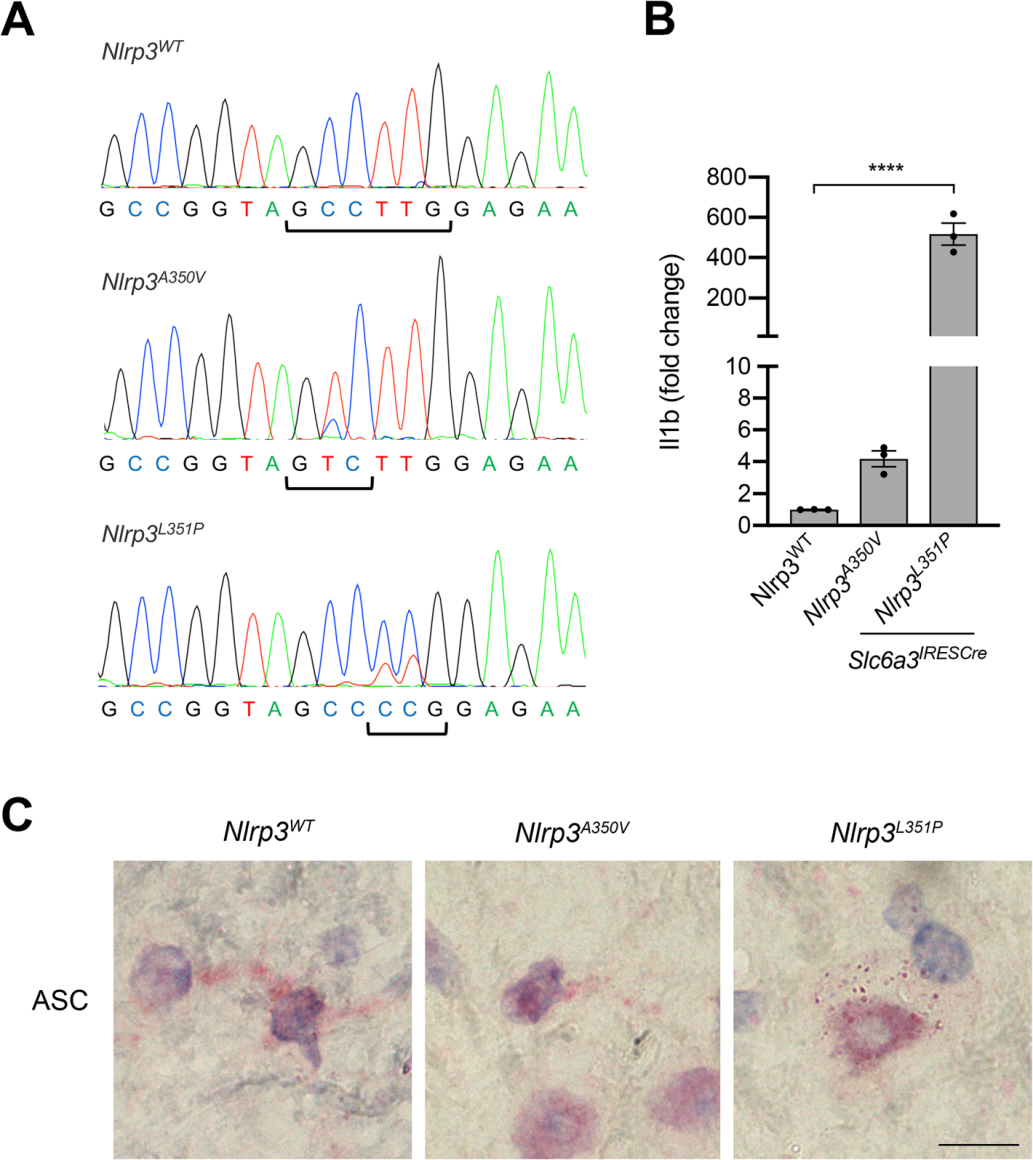
Mutant alleles expressed in striatal tissues, evidence of NLRP3 inflammasome activity at 18 months of age. **a** mRNA was isolated from striatal tissues, reverse transcribed, amplified, and sequenced to confirm expression of *Nlrp3* allele in target tissues. The alanine to valine substitution at aa350 was observed in mice expressing *Nlrp3*^*A350V*^ (C➔ T, red peak), with evidence of the *Nlrp3*^*WT*^ background copy expression (blue peak). The leucine to proline substitution at aa351 was observed in mice expressing *Nlrp3*^*L351P*^ (TT ➔ CC, blue peaks), with evidence of the *Nlrp3*^*WT*^ background copies expressed (red peaks). **b***Il1b* mRNA transcript was reverse transcribed, amplified, and quantified with real-time PCR technologies. *Nlrp3*^*L351P*^ mice expressed significantly more *Il1b* mRNA in striatal tissues compared to control animals (**** *p* < 0.0001, one-way ANOVA, error bars represent s.e.m.). **c** Histologic sections of substantia nigra were probed with anti-ASC antibodies, followed by BOND polymer detection, and imaged with a brightfield microscope. ASC puncta (red) indicative of NLRP3 inflammasome activity were observed in *Nlrp3*^*L351P*^ mice. (60 × images, scale bar represents 10 μM)

Following the observation that *Nlrp3*^*L351P*^ animals exhibit an age-related reduction in motor function, we conducted post-mortem analysis of brain tissues to characterize NLRP3 inflammasome activity, DA neuron cell number, and neuroinflammation in our cohorts. We confirmed expression of *Nlrp3* mutant transcripts using mRNA isolated from striatal tissues at 18 months of age. The hemizygous animal model is complex, harboring either wild-type *Nlrp3* alleles alone or a wild-type *Nlrp3* allele in combination with *Nlrp3*^*A350V*^ or *Nlrp3*^*L351P*^ alleles in *Slc6a3*-expressing cells. We isolated mRNA from dopamine fiber-rich striatal tissues and PCR amplified cDNA sequences flanking the region of *Nlrp3* engineered to contain the CAPS-associated mutations. Direct sequencing of these *Nlrp3* amplicons demonstrated expression of both wild-type *Nlrp3* mRNA and transcripts containing *Nlrp3*^*A350V*^ or *Nlrp3*^*L351P*^ mutations in striatal tissues (Fig. 3a), consistent with planned hemizygosity (Fig. 1a, b), and the findings described in the original manuscript characterizing these mice [48]. To assess enhanced activity of NLRP3 in animals expressing *Nlrp3*^*A350V*^ or *Nlrp3*^*L351P*^, we evaluated well-characterized and histopathologically identifiable ASC-immunoreactive specks, whose presence is indicative of inflammasome activation [60]. Immunostaining of mesencephalic sections with anti-ASC antibodies revealed distinct ASC-immunoreactive puncta in the substantia nigra pars compacta (SNpC) of animals expressing *Nlrp3*^*L351P*^ that were not readily observed in animals expressing *Nlrp3*^*WT*^ and *Nlrp3*^*A350V*^ (Fig. 3c). In addition to the formation of ASC immunoreactive puncta, we measured the relative expression of *Il1b*, whose elevation is indicative of an inflammasome-driven proinflammatory response [1]. Real-time PCR experiments revealed elevated *Il1b* transcript in striatal tissues from both mutant animals, with significantly elevated expression in animals expressing *Nlrp3*^*L351P*^ (Fig. 3b). These findings are consistent with the original report that described more severe phenotype associated with the *Nlrp3*^*L351P*^ allele as compared with the *Nlrp3*^*A350V*^ allele [48]. Importantly, the presence of *Slc6a3*-driven *Nlrp3*^*A350V*^ and *Nlrp3*^*L351P*^ transcripts in the aged mesencephalon is consistent with our studies in PD patients [40] indicating expression of the endogenous *Nlrp3* locus in mesencephalic neurons.

**Fig. 4:**
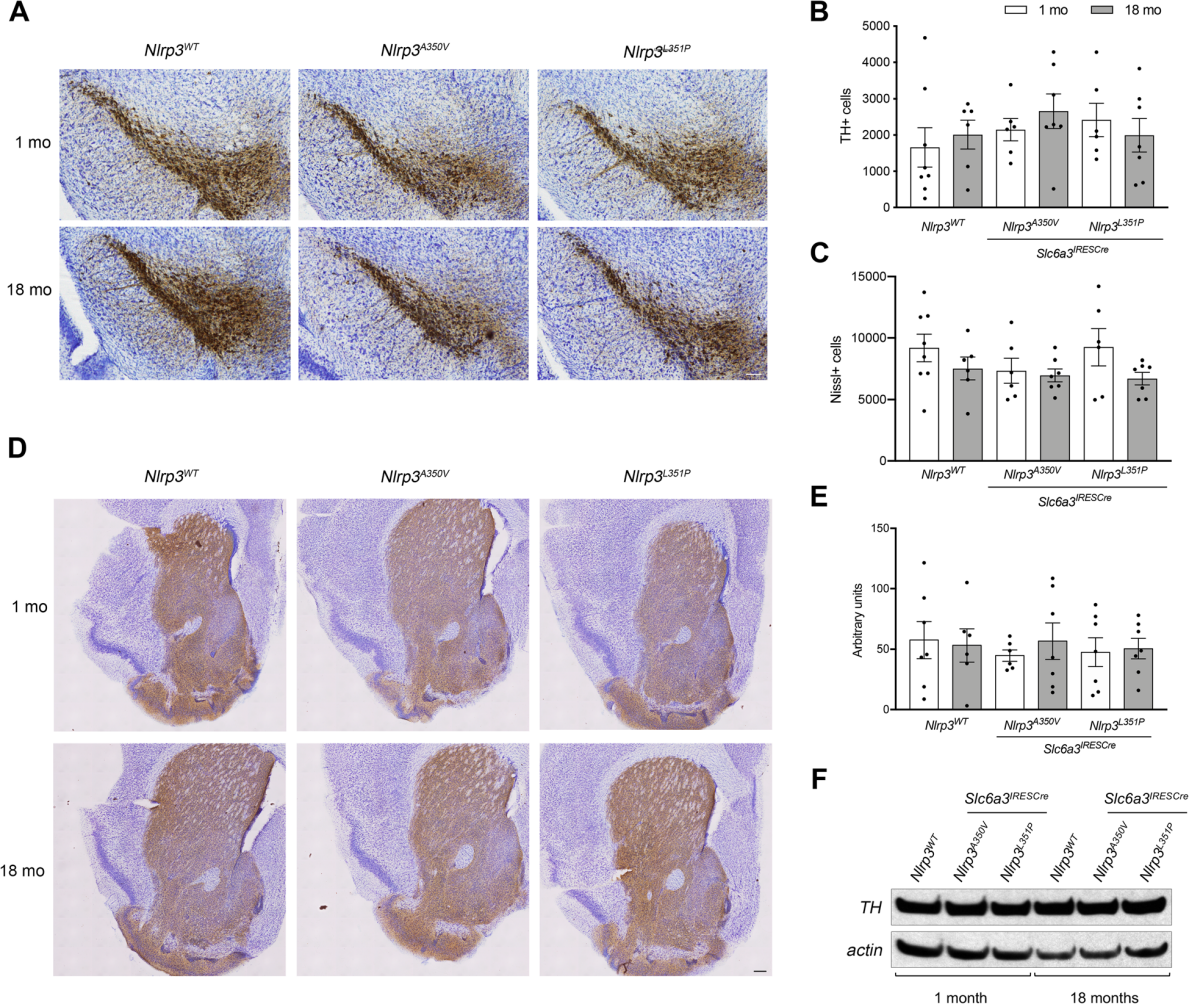
Analysis of DA neuron maintenance in SNpC and striatal tissues from young and old mice. **a** Mesencephalic sections were stained with anti-tyrosine hydroxylase antibodies and counterstained with Nissl. TH and Nissl positive cells were counted in sections obtained from 1- and 18-month-old animals. Scale bar represents 100 μM. **b** Quantification of TH-immunoreactive neurons in the SNpC of animals expressing *Nlrp3*^*WT*^, *Nlrp3*^*A350V*^, and *Nlrp3*^*L351P*^. No significant differences in TH-immunoreactive cells were observed among cohorts. **c** Quantification of Nissl-positive neurons in the SNpC. No significant differences were observed with one-way ANOVA analysis. Error bars represent s.e.m. **d** Representative sections of striatal tissues from 1- and 18-month-old animals were sectioned and stained with anti-TH antibodies. Scale bar represents 500 μM. **e** Densitometric analysis of TH staining in the striatum revealed no significant differences among genotypes and ages with one-way ANOVA analysis. Error bars represent s.e.m. **f** Striatal tissues were homogenized and proteins were separated by SDS-PAGE. Immunoblotting with anti-TH antibodies was conducted. No significant differences in TH expression among genotypes or with age were observed. Anti-actin antibodies were used as a loading control

To determine if expression of mutant *Nlrp3* alleles resulting in hyperactive NLRP3 protein affected the maintenance of DA neurons throughout aging, we conducted unbiased stereology at 1- and 18-month time points in a single brain hemisphere (see “Methods” section) (Fig. 4a). One-way ANOVA analysis did not reveal significant changes in the number of TH-immunoreactive and Nissl-positive cells present in the SNpC of these tissues (Fig. 4b, c). Histologic analysis of striatal tissues immunostained with anti-TH antibodies and western blot analysis of striatal tissue homogenates revealed no significant change in TH expression (Fig. 4d–f), confirming our findings that while variable, there is no genotype-specific change in the number of DA neurons in the substantia nigra in our mouse cohorts.

**Fig. 5:**
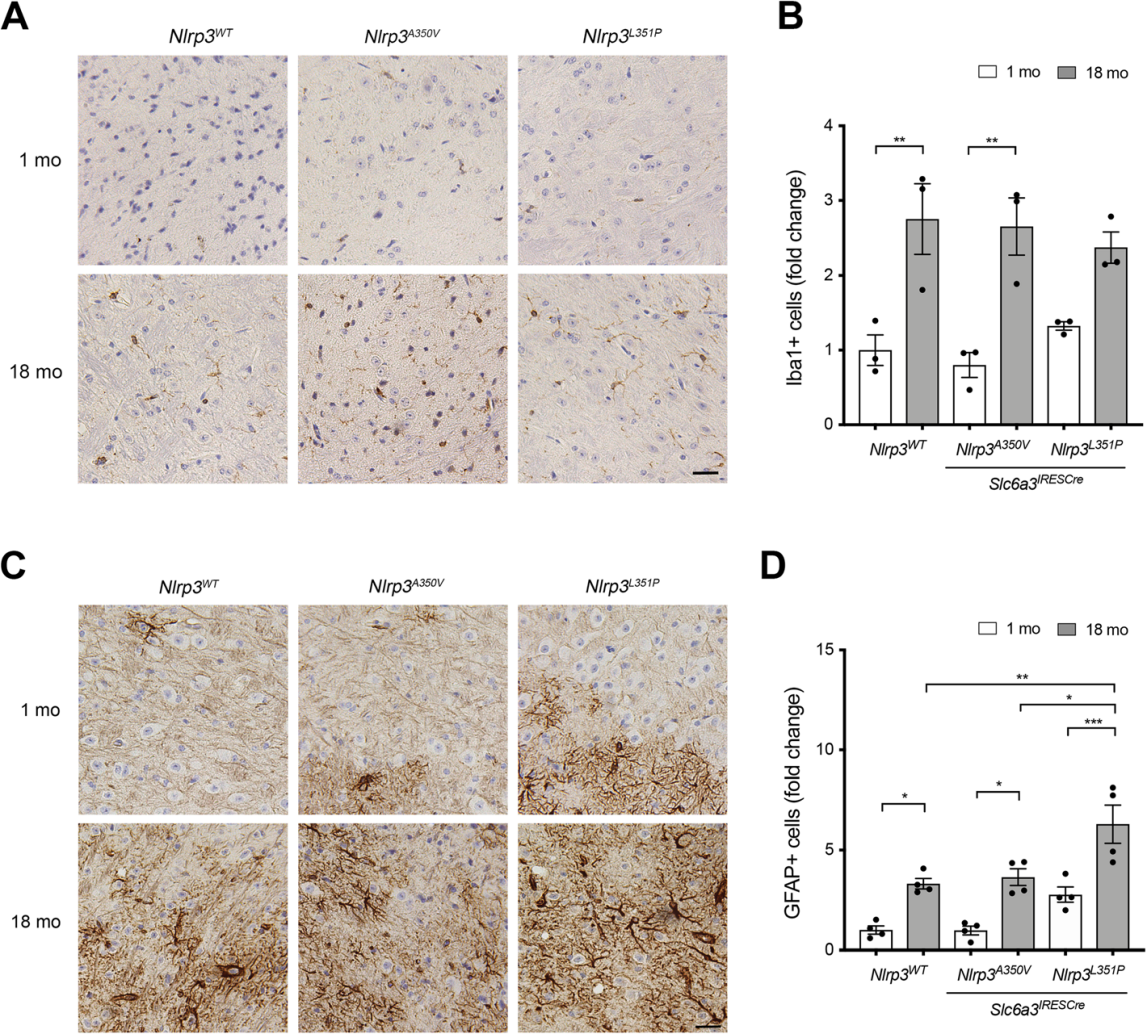
Increased number of IBA1-immunoreactive and GFAP-immunoreactive cells in the SNpC of 18-month-old animals. **a** Representative histologic sections of the SNpC obtained from 1- and 18-month-old animals stained with anti-IBA1 antibodies and counterstained with BOND polymer detection. **b** IBA1-immunoreactive cells were quantified (10 fields/animal, *n* = 3). The number of IBA1-immunoreactive cells was significantly greater in 18-month-old animals compared to 1-month-old animals, indicating number of microglia in the SNpC increases with age. (*Nlrp3*^*WT*^ ** *p* = 0.0094, one-way ANOVA; *Nlrp3*^*A350V*^ ** *p* = 0.0041, one-way ANOVA). **c** Representative IHC images of SNpC tissues stained with anti-GFAP antibodies followed by BOND polymer detection. **d** GFAP-immunoreactive cells were quantified (10 fields/animal, *n* = 4). GFAP-immunoreactive cell number increases with age in all cohorts (*Nlrp3*^*WT*^ * *p* = 0.03, *Nlrp3*^*A350V*^ * *p* = 0.01, *Nlrp3*^*L351P*^ *** *p* = 0.0008, one-way ANOVA). The number of GFAP-immunoreactive cells was significantly greater in 18-month-old *Nlrp3*^*L351P*^ animals compared to the number of cells in 18-month-old *Nlrp3*^*WT*^ and *Nlrp3*^*A350V*^ animals (** *p* = 0.004 and * *p* = 0.01 respectively, one-way ANOVA). Scale bars represent 20 μM. Error bars represent s.e.m.

Inflammatory signaling from neurons is anticipated to be closely monitored by glial cell populations in the brain microenvironment [61, 62]. We characterized microglia and astrocytes in nigrostriatal brain tissues from 1- and 18-month-old animals to determine if neuronal expression of the mutant *Nlrp3* alleles influenced glia throughout aging. Quantification of IBA1-immunoreactive microglia cells in SNpC tissues from 1- and 18-month-old animals (Fig. 5a) revealed a significant increase in cell number with age in all three genotypes (Fig. 5b). We also quantified the number of ramified and activated microglia in these tissues and observed a similar pattern with no differences between genotypes (data not shown). Staining adjacent sections with anti-GFAP antibodies to characterize astrocytes (Fig. 5c), we observed a significantly greater number of GFAP-immunoreactive astrocytes in the SNpC in 18-month-old animals compared to 1-month-old animals in all genotypes (Fig. 5d). In addition to observing an increased number of GFAP-immunoreactive astrocytes associated with aging, we also observed significantly more astroglia in 18-month-old animals expressing *Nlrp3*^*L351P*^ compared to animals expressing *Nlrp3*^*WT*^ and *Nlrp3*^*A350V*^. We and others have previously reported astrocytes to be a direct target of IL-1β [14, 63–65], the canonical proinflammatory effector cytokine of the inflammasome [7], and others have previously reported evidence of neuronal IL-1β expression [66]. Analysis of SNpC tissues was consistent with these previous reports, demonstrating easily observable IL-1β expression in TH-immunoreactive fibers (Supplementary Figure 2). While we did not observe significant nigral degeneration, these data support a model in which endogenous activity of the NLRP3 inflammasome, enhanced by the presence of the *Nlrp3*^*L351P*^ allele, is associated with age-dependent astrogliosis and behavioral deficits.

**Fig. 6:**
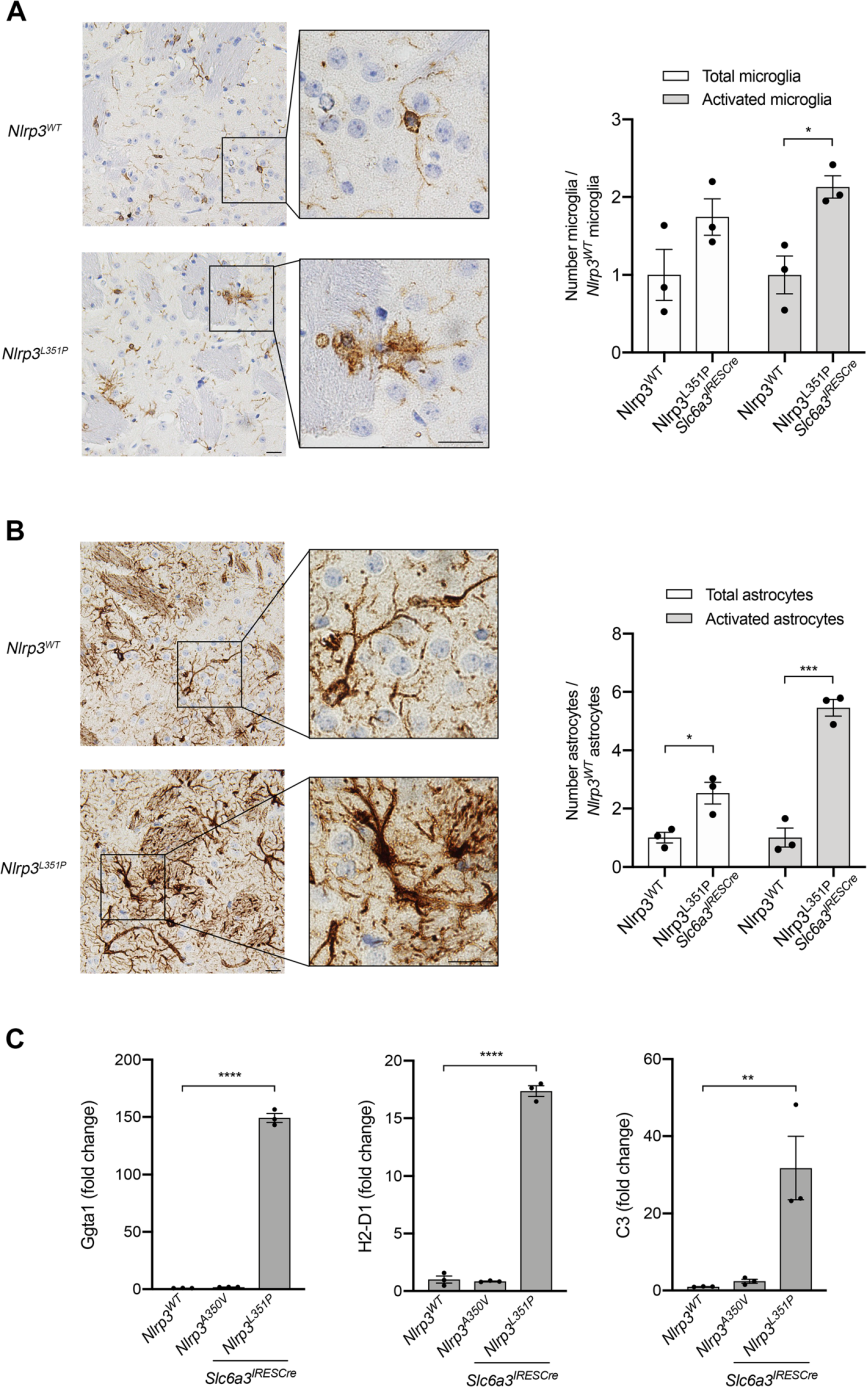
Evidence of enhanced proinflammatory glial cell phenotype in the striatum of *Nlrp3*^*L351P*^ animals. **a** Striatal tissue sections from 18-month-old animals were probed with anti-IBA1 antibodies followed by BOND polymer detection. Microglial activation can be visualized in animals harboring the *Nlrp3*^*L351P*^ allele (bottom panel), compared to their WT littermates (top panel). Scale bars represent 20 μM. There are significantly more activated microglia in tissues from animals expressing *Nlrp3*^*L351P*^ compared with tissues from *Nlrp3*^*WT*^ animals (* *p* = 0.0164, *t* test). Error bars represent s.e.m. **b** Adjacent striatal sections were stained with anti-GFAP antibodies. GFAP-immunoreactive astrocytes in *Nlrp3*^*L351P*^ animals have an increased activation phenotype (bottom panel), compared to *Nlrp3*^*WT*^ animals (top panel). Scale bars represent 20 μM. Quantitation revealed significantly more total astrocytes (* *p* = 0.0217, *t* test) as well as significantly more activated astrocytes (*** *p* = 0.0005, *t* test) in striatal tissues from *Nlrp3*^*L351P*^ animals compared to controls. Error bars represent s.e.m. **c** Expression of A1 astrocyte markers, *Ggta1*, *H2-D1*, *C3*, were assessed in 18-month-old animals. RNA was isolated from striatal tissues, reversed transcribed, and mRNA was measured by real-time PCR. *Ggta1*, *H2-D1*, and *C3* levels were significantly elevated in *Nlrp3*^*L351P*^ mice compared to *Nlrp3*^*WT*^ animals (**** *p* < 0.0001, **** *p* < 0.0001, ** *p* = 0.009 respectively, one-way ANOVA). Error bars represent s.e.m.

Based on our observation of IL-1β expression in neuronal fibers, we evaluated striatal tissues, reasoning that proinflammatory changes induced by DA neurons may be evident in this fiber-rich projection site. Unlike in the SNpC, microglia activation was easily observable in tissues obtained from animals expressing *Nlrp3*^*L351P*^ (Fig. 6a, bottom panel) compared with tissues obtained from *Nlrp3*^*WT*^ animals (top panel). Similarly, GFAP immunostaining indicated the presence of supernumerary astrocytes with a proinflammatory morphology in *Nlrp3*^*L351P*^ animals (Fig. 6b, bottom panel), which was not observed in *Nlrp3*^*WT*^ expressing animals (top panel). To further confirm our observation of proinflammatory changes in astroglial phenotype in both SNpC (Fig. 5c, d) and striatal tissues (Fig. 6b), we isolated mRNA from freshly frozen striatal tissues obtained from 18-month animals and measured expression of proinflammatory A1 astrocyte markers. We observed elevated expression of *Ggta1*, *H2-D1*, and *C3* in animals expressing *Nlrp3*^*L351P*^ compared to animals expressing *Nlrp3*^*WT*^ and *Nlrp3*^*A350V*^ (Fig. 6c). These results provide evidence of microglial activation (Fig. 6a) and the presence of proinflammatory astrocytes in animals expressing the *Nlrp3*^*L351P*^ activating mutation in DA neurons (Fig. 6b, c), suggesting that in the aging brain, DA neurons have the capacity to initiate a widespread *Nlrp3*-dependent neuroinflammatory response associated with progressive loss of motor function in the absence of neuronal cell death.

## Discussion

Numerous reports support a role for inflammasomes functioning in CNS microglia during normal aging and the progression of age-related neurodegenerative disorders [16, 19, 33, 34]. Our studies have been consistent with these reports [14]; however, we also unexpectedly identified *NLRP3* expression in a subset of surviving DA neurons in late-stage PD patients and neuronal cell models [40]. Others report inflammasome activity in neurons [27, 53–55], but to our knowledge, no in vivo studies have examined directly the function of NLRP3 in DA neurons in the context of aging. In this study, we generated mice with the potential for DA neuron-specific NLRP3 inflammasome hyperactivity and analyzed the animals throughout aging (Fig. 1). The experiments indicate that mesencephalic neurons express the endogenous *Nlrp3* gene, and that modification of the endogenous *Nlrp3* locus to mimic a CAPS-associated hyperactive allele results in progressive motor dysfunction and nigrostriatal inflammation.

Among neuronal cell types, DA neurons represent an intuitive host for activity of intracellular danger sensing inflammasome signaling pathways. The processes of dopamine synthesis and metabolism induce cellular stress, including the production of dopamine quinones and intracellular ROS, a frequently reported trigger of the NLRP3 inflammasome [67, 68]. Oxidized dopamine molecules are capable of altering DNA and proteins [68], as well as inducing mitochondrial and lysosomal dysfunction, highly penetrant molecular phenotypes associated with both familial and sporadic PD [69] and reminiscent of sterile inflammatory triggers known to activate inflammasomes. These unique biologies may underlie selective vulnerability of DA neurons [70]; chronic basal levels of cellular stress are thought to negatively impact their survival throughout aging and in circumstances of toxicant-exacerbated distress. A relationship between dopamine and the NLRP3 inflammasome has been previously identified by Yan and others who report suppression of NLRP3 inflammasome-driven inflammation dependent on the DRD1 dopamine receptor in bone marrow-derived macrophages [71]. In a related report, astroglial DRD2 receptors are similarly implicated in suppression of NLRP3 inflammasome activity [72]. Critically, DA neurons express DRD2 autoreceptors that are involved in regulating the synthesis, release, and uptake of dopamine in presynaptic cells [73]. Dysregulation of these receptors is associated with multiple neurologic disorders including PD [74]. These reports highlight a regulatory pathway in which inflammasome activity is modulated by dopamine receptor activity, possibly as a means of maintaining homeostasis in microenvironments containing vulnerable neuronal populations. As animals age, dopamine receptor expression has been reported to decrease [75], which would alter the negative regulation of NLRP3-mediated inflammation. Our findings are consistent with this relationship, as we observed increased activity of the endogenous *Nlrp3* locus in older animals, resulting in neuroinflammation exacerbated by the presence of the L351P NLRP3 activating mutation (Figs. 5 and 6). Findings that expression of hyperactive *Nlrp3* alleles can tip the balance towards nigrostriatal inflammation and motor deficits at advanced age frame the question of whether genetic and/or environmental triggers could further upset this balance triggering sustained neurogenic inflammation capable of activating astrocytes and microglia within the nigrostriatal microenvironment.

In our study, we identified motor abnormalities specific to the L351P NLRP3 mutation in the absence of nigral cell loss. In this context, we observed changes in glial populations indicating astroglial activation in both the substantia nigra and the striatum, as well as microglial activation in striatal tissues (Figs. 5 and 6). Changes in astrocyte phenotype may help to reconcile the finding of motor deficits in animals with no significant loss of dopaminergic neurons (Figs. 2 and 4). Perisynaptic astroglia are critical for neurotransmission, forming tripartite synapses that include pre- and post-synaptic neurons [76]. Astrocyte activation, observed in conditions including stroke [77] and neurodegeneration, alters synaptic function and neurotransmission [78]. Therefore, it stands to reason that dopamine transmission in the striatum may be altered in concert with the pronounced proinflammatory astrocyte activation we observe in the *Nlrp3*^*L351P*^ mice (Fig. 6) and thereby contribute to synaptic inefficiency and motor deficits. This possibility is supported by our finding of less severe neuroinflammatory and behavioral deficits in *Nlrp3*^*A350V*^ animals despite similar numbers of nigral neurons (Fig. 4). Our findings are also reminiscent of PD patients who have clinical manifestations of PD based on motor symptomology, but upon imaging analysis has no observable loss of dopaminergic neurons [79]. This patient population has been referred to as “Scans Without Evidence for Dopaminergic Deficit (SWEDD).” A plausible prediction is that we have modeled the earliest stages of motor abnormalities resulting from neuronal distress associated with widespread inflammation and that analysis of animals at more advanced ages could reveal neurodegeneration.

Recently, there is an increasing appreciation for the role of astroglia in PD [80]. We observed specific changes in numbers of GFAP-immunoreactive astrocytes in the SNpC of animals expressing *Nlrp3*^*L351P*^, compared with animals expressing *Nlrp3*^*WT*^ and *Nlrp3*^*A350V*^ (Fig. 5). However, it is important to note that loss of animals in our *Nlrp3*^*A350V*^ cohort reducing the group size to six may have impacted our statistical power. Astroglial changes were more pronounced in animals expressing *Nlrp3*^*L351P*^, observable in both the SNpC and the striatum, compared to phenotypically activated IBA1-immunoreactive microglia, which were only observed in the striatum (Fig. 6). To more closely examine these astrocytes, we measured mRNA expression and observed evidence of an enhanced proinflammatory A1 astrocyte signature only in *Nlrp3*^*L351P*^ animals (Fig. 6). Previous reports of glial biology in PD patient brains report evidence of astrogliosis in the SNpC, and many of the PD genes (“PARK genes”) are known to be important in maintaining cellular processes in astrocytes, as well as in neurons [80]. The temporal function of astroglia within the processes of PD-associated neuroinflammation and neurodegeneration remains unclear. The majority of evidence suggests that astrocytes become activated subsequent to microglial activation [81]; however, it has also been shown that astrocytes can become activated in the absence of microglia-specific cytokines [82]. It is plausible that in our model, distressed DA neurons harboring elevated NLRP3 inflammasome activity communicate directly with astrocytes, consistent with the reported neuroprotective role of astroglia. We posit that at advanced ages in contexts of elevated cellular stress modeled in this study with NLRP3-activating mutations, the neuroprotective function of astrocytes is unable to persist, and ultimately the proinflammatory astroglial phenotype triggers microglial activation (Figs. 5 and 6). Our decision to analyze mice retaining a wild-type *Nlrp3* allele in all cell types creates both opportunities and confounds. As a result, it is impossible to distinguish NLRP3 inflammasome activity directly related to inclusion of the hyperactivated transgene from wild-type NLRP3 inflammasome activity in glia resulting from the neuronal perturbation. For this reason, we are unable to determine with certainty that the *Nlrp3*^*L351P*^ allele was not expressed inappropriately in glia, although we believe this to be unlikely due to previous studies utilizing this strain and our characterization of microglia and astrocytes [49–52]. Despite the shortcomings of this model system, the inclusion of an endogenous *Nlrp3* allele was likely a critical factor in our discovery of an expanding neuroinflammatory phenotype resulting from expression of the *Nlrp3*^*L351P*^ allele of neurons, a finding of importance as we work to understand the earliest stages of neuroinflammation in neurodegenerative disease. Further experiments to directly test the function of neuronal NLRP3, as well as astrocyte and microglia-specific activation in similar model systems, will more clearly characterize these important neuroglia interactions. Such studies will continue to be of high interest as they may help identify novel neuroprotective targets operating at the earliest stages of inflammatory neurodegenerative disorders.

## Conclusions

Neuroinflammation is pathologically associated with many neurodegenerative disorders. We recently reported expression of NLRP3 in dopaminergic neurons in late-stage PD patient tissue [40] and have in vivo evidence that loss of *Nlrp3* may prevent the progression of pathology in a toxicant model of PD [14]. Here, we developed a longitudinal mouse model to specifically determine the impact of neuronal NLRP3 activity in aging. Findings from this study conclude that aberrate NLRP3 function in DA neurons, due to the presence of *Nlrp3*-activating mutations associated with the CAPS diseases, results in motor function deficit and proinflammatory astrogliosis. These results are significant because they highlight complex distress-related neuroglial processes and provide a model of molecular events that exist at the transition from tissue homeostasis to progressive degenerative disease.

## Supplementary information

**Additional file 1: **Supplementary Figure 1. Open field analysis characterizing motor function throughout longitudinal study. Behavioral analysis of mice expressing *Nlrp3*^*WT*^ (n = 11), *Nlrp3*^*A350V*^ (n = 6), and *Nlrp3*^*L351P*^ (n = 9) was conducted at 6, 10, 14 and 18-month time-points. (A) Mice expressing *Nlrp3*^*L351P*^ became significantly less mobile over time (two-way ANOVA, p = 0.0018 (genotype), p < 0.0001 (time); Tukey’s post hoc multiple comparisons test, ** p = 0.0027) and (B) had a significant reduction in average velocity (two-way ANOVA, p = 0.0008 (genotype), p < 0.0001 (time); Tukey’s post hoc multiple comparisons test, ** p = 0.0020). (C) Older animals of all genotypes exhibited significant changes in amount of time spent in the center of the testing box over the time-course of the longitudinal study (two-way ANOVA, p = 0.0319 (time)). Error bars represent s.e.m.

**Additional file 2: **Supplementary Figure 2. IL-1β protein detected in TH-immunoreactive axons. Histologic section of SNpC tissues from 18-month old animals were stained with anti-IL-1β and anti-TH antibodies. (A) Confocal microscopy of tissues from *Nlrp3*^*L351P*^ animals revealed IL-1β protein expression in TH- immunoreactive axons (bottom panels) but not in TH-immunoreactive cell bodies (top panels). Scale bar represents 20 μM. (B) Colocalization of IL-1β and TH was observed in histologic sections from all three genotypes. Scale bar represents 10 μM.

## Data Availability

Data sharing is not applicable as no datasets were generated or analyzed in this study.

## Abbreviations

AD: Alzheimer’s disease
ALR: Absent in melanoma 2-like receptor
ANOVA: Analysis of variance
ASC: Apoptosis-associated speck-like protein containing a CARD
CAPS: Cryopyrin-associated periodic syndromes
CNS: Central nervous system
DA: Dopaminergic
FCAS: Familial cold autoinflammatory syndrome
GFAP: Glial fibrillary acidic protein
HRP: Horse radish peroxidase
IBA1: Ionized calcium-binding adaptor molecule 1
IL-1β: Interleukin-1 beta
LUHMES: Lund human mesencephalic cells
MWS: Muckle-Wells syndrome
NLR: Nucleotide-binding domain-like receptor
Nlrp3: NACHT, LRR, and PYD domains-containing protein 3
NOMID: Neonatal onset multisystem inflammatory disease
PARK genes: Parkinson’s associated genes
PD: Parkinson’s disease
ROS: Reactive oxygen species
SNpC: Substantia nigra pars compacta
SWEDD: Scans without evidence for dopaminergic deficit
TH: Tyrosine hydroxylase
