# Supplementary figures and images for: Slc6a3-dependent expression of a CAPS-associated Nlrp3 allele results in progressive behavioral abnormalities and neuroinflammation in aging mice
Source: J Neuroinflammation. 2020 Jul 17;17:213. doi: 10.1186/s12974-020-01866-6 (PMC7368774; doi:10.1186/s12974-020-01866-6)

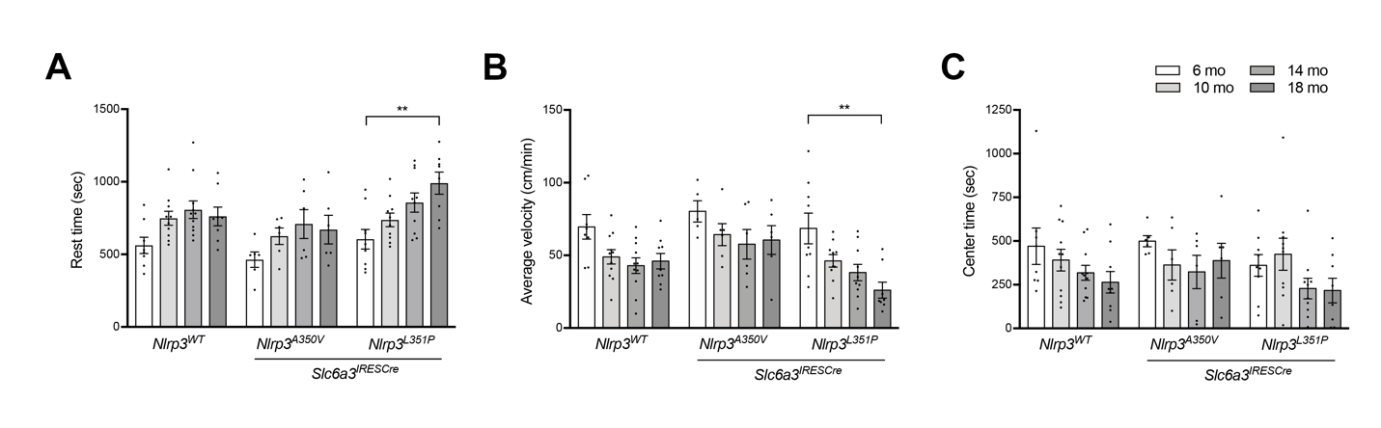

Supplement: Supplementary file 1 — Additional file 1: Supplementary Figure 1. Open field analysis characterizing motor function throughout longitudinal study. Behavioral analysis of mice expressing Nlrp3WT (n = 11), Nlrp3A350V (n = 6), and Nlrp3L351P (n = 9) was conducted at 6, 10, 14 and 18-month time-points. (A) Mice expressing Nlrp3L351P became significantly less mobile over time (two-way ANOVA, p = 0.0018 (genotype), p < 0.0001 (time); Tukey’s post hoc multiple comparisons test, ** p = 0.0027) and (B) had a significant reduction in average velocity (two-way ANOVA, p = 0.0008 (genotype), p < 0.0001 (time); Tukey’s post hoc multiple comparisons test, ** p = 0.0020). (C) Older animals of all genotypes exhibited significant changes in amount of time spent in the center of the testing box over the time-course of the longitudinal study (two-way ANOVA, p = 0.0319 (time)). Error bars represent s.e.m. [file 12974_2020_1866_MOESM1_ESM.docx]

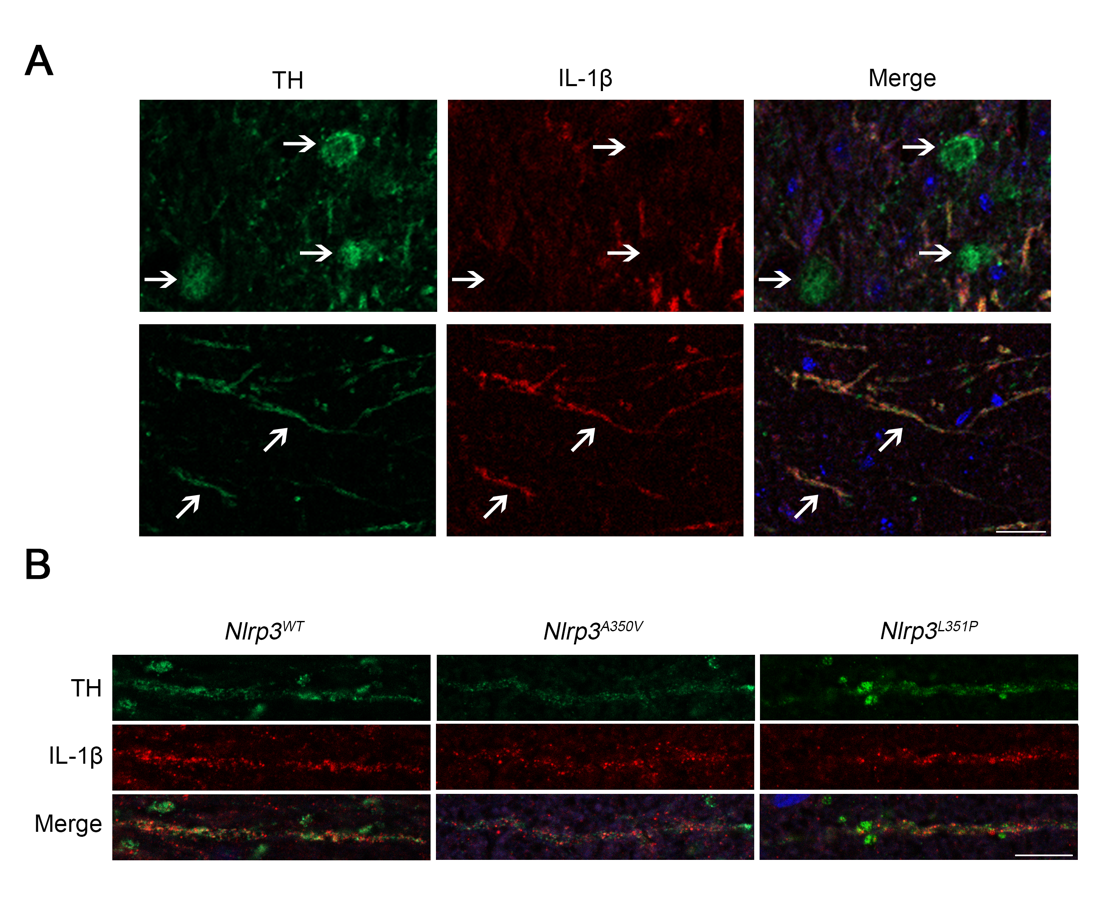

Supplement: Supplementary file 2 — Additional file 2: Supplementary Figure 2. IL-1β protein detected in TH-immunoreactive axons. Histologic section of SNpC tissues from 18-month old animals were stained with anti-IL-1β and anti-TH antibodies. (A) Confocal microscopy of tissues from Nlrp3L351P animals revealed IL-1β protein expression in TH- immunoreactive axons (bottom panels) but not in TH-immunoreactive cell bodies (top panels). Scale bar represents 20 μM. (B) Colocalization of IL-1β and TH was observed in histologic sections from all three genotypes. Scale bar represents 10 μM. [file 12974_2020_1866_MOESM2_ESM.docx]
